# Multidimensional influencing factors of postpartum depression based on the perspective of the entire reproductive cycle: evidence from western province of China

**DOI:** 10.1007/s00127-024-02686-2

**Published:** 2024-05-24

**Authors:** Yiyun Zhang, Xinwei Liu, Mengmei Liu, Min Li, Ping Chen, Guanghong Yan, Qingyan Ma, Ye Li, Dingyun You

**Affiliations:** 1 School of Ethnology and Sociology, Yunnan University, Kunming, 650091 Yunnan China; 2 Research Center of Health Policy and Management, School of Health Management, Harbin Medical University, Harbin, 150086 Heilongjiang China; 3 School of Public Health, Kunming Medical University, Kunming, 650500 Yunnan China; 4 School of Public Health, Hangzhou Normal University, Hangzhou, 311121 Zhejiang China; 5 Yunnan Provincial Key Laboratory of Public Health and Biosafety & School of Public Health, Kunming Medical University, Kunming, 650500 Yunnan China

**Keywords:** Postpartum depression, Risk factors, Family support, Social support

## Abstract

**Objective:**

China has a serious burden of Postpartum depression (PPD). In order to improve the current situation of high burden of PPD, this study explores the factors affecting PPD from the multidimensional perspectives with physiology, family support and social support covering the full-time chain of pre-pregnancy–pregnancy–postpartum.

**Methods:**

A follow-up survey was conducted in the Qujing First People’s Hospital of Yunnan Province from 2020 to 2022, and a total of 4838 pregnant women who underwent antenatal checkups in the hospital were enrolled as study subjects. Mothers were assessed for PPD using the Edinburgh Postnatal Depression Scale (EPDS), and logistic regression was used to analyse the level of mothers’ postnatal depression and identify vulnerability characteristics.

**Results:**

The prevalence of mothers’ PPD was 46.05%, with a higher prevalence among those who had poor pre-pregnancy health, had sleep problems during pregnancy, and only had a single female fetus. In the family support dimension, only family care (OR = 0.52, 95% CI 0.42–0.64) and only other people care(OR = 0.78, 95% CI 0.64–0.96) were the protective factors of PPD. The experience risk of PPD was higher among mothers who did not work or use internet.

**Conclusion:**

The PPD level in Yunnan Province was significantly higher than the global and Chinese average levels. Factors affecting mothers’ PPD exist in all time stages throughout pregnancy, and the influence of family support and social support on PPD shouldn’t be ignored. There is an urgent need to extend the time chain of PPD, move its prevention and treatment forward and broaden the dimensions of its intervention.

## Introduction

Postpartum depression (PPD) is caused by a combination of factors in the pre-pregnancy, pregnancy, and postpartum periods. It is one of the most prevalent and disabling but most underappreciated complications in women of childbearing age [1]. PPD not only leads to mothers’ morbidity in forms of guilt, fatigue, loss of appetite and sleep disorder. It also adversely affects the health and well-being of the newborn, partners and other family members, disrupting infant care and family dynamics [2]. PPD is experienced by women around the world, making it an important public health issue [3]. Globally, about 10–15% of the mothers suffered PPD in the postpartum period, while the prevalence of PPD was about 9.4% to 27.4% in China [4]. PPD is not only affected by physiological factors such as changes in hormone and immune levels but also affected by traditional Chinese fertility culture [5]. Chinese mothers are subject to higher levels of family and social intervention. Moreover, 40% of women who have experienced PPD will experience depression again in their lifetime; nearly 50% will experience PPD again in subsequent pregnancies [6].

Mothers are vulnerable to depression due to a combination of psychological and social attributes [7, 8]. In the case of depression, mothers’ treatment options are limited by the potential adverse effects of medications on the baby [9]. Therefore, early preconception identification, intervention and elimination of risk factors for PPD are particularly important for both mothers and newborns. However, previous studies have mostly focused on capturing risk factors for mothers’ depression during pregnancy and postpartum [10–13], without fully considering the time-cumulative characteristics of PPD, lacking analyses of factors such as pre-pregnancy health and pre-pregnancy preparation, and the nodes of concern have not covered the full chain of the pregnancy cycle. It was also found that previous studies have explicitly explored the mechanism of physiological factors influencing PPD [14, 15], and although some studies have also paid attention to the impact of multidimensional factors such as family support and social risk on PPD [16, 17], the mechanisms of influence remain unclear.

Generally, this study takes pre-pregnancy, pregnancy and post-pregnancy as analysis nodes to identify the vulnerability gap of PPD in the whole-time chain, comprehensively considers the multi-attribute characteristics of the disease, and explores the influencing mechanism of PPD based on multiple dimensions of socio-demographic, family support and social support.

## Methods

### Study sample

The study subjects were pregnant women undergoing prenatal care at Qujing First People’s Hospital Inclusion Criteria: have reading comprehension and communication skills, voluntarily participate in the survey and complete the questionnaire. Exclusion criteria: stillbirth, birth defects, miscarriage, lack of information, etc. The total of 4838 participants were included in this study after deleting missing values and abnormal values.

### Questionnaire and content

A self-designed questionnaire was used to collect demographic data on pregnant women and information on depression during postpartum. The demographic data of pregnant women include: residence, marital status, pre-pregnancy health status, whether trained in pregnancy knowledge, whether the pregnancy reaction is intense, single or double fetus, sleep, gestational weeks of childbirth, depression score, infant gender, infant birth weight, type of care, work, and Internet use.

### Screening tools for PPD

Depressive symptoms are assessed using the EPDS, which is widely used to screen for PPD. The scale assesses the intensity of depressive symptoms in the past 7 days, and contains a total of 10 items, each of which is divided into “never”, “occasionally”, “often”, and “always” according to the intensity of depressive symptoms, corresponding to “0 points”, “1 point”, “2 points”, and “3 points”. The total score ranges from 0 to 30 points, and the higher the score, the more severe the depressive symptoms. In this study, participants were divided into depressive (≥ 12) and non-depressive (< 12) groups using 12 points as cut-off values.

### Model variable

The dependent variable in this study was PPD.

The covariates included in this study were socio-demographic factors (residence and marital status), pre-pregnancy factors (pre-pregnancy health status and whether trained in pregnancy knowledge), pregnancy factors (whether the pregnancy reaction is intense, single or double fetus, sleep, and gestational weeks of childbirth), postpartum factors (gender of the infant, birth weight of the infant), family support factors (type of care), and social support factors (work and internet use).

### Statistical analysis

The study used SPSS 22.0 for descriptive statistics analysis. Firstly, each risk factor was individually tested for variability. Secondly, using logistic regression model, all significant risk factors were subjected to multivariate logistic regression, in order to assess the risk factors for PPD. The results of the study were expressed as odds ratio (OR) and 95% confidence interval (CI). A p-value of less than 5% was considered statistically significant in all tests.

## Results

### Sample characteristics

Table 1 presents the basic information of all participants. More than half of the participants lived in the city (67.16%); participants’ marital status was mostly married (88.90%). 92.35% of the participants reported having good pre-pregnancy health conditions; the percentage of attending pregnancy knowledge training was relatively balanced, with 58.60% of participants attending and 41.40% not attending. In the pregnancy period, about one-third of the participants reported that they had severe pregnancy reactions (34.15%); only a small percentage of the mothers gave birth to double fetuses (1.84%); the percentage of the participants who had normal sleep was 59.1%, while 18.44% often insomnia or sleep poorly and 22.53% somnolence; and the majority of the mothers gave birth at full term (93.12%). In the postpartum period, the prevalence of PPD was 46.05%, single fetus only with the gender of the infant was female or male was more prevalent in the variable of the infant gender, their values respectively were 47.15% and 52.07%, and the majority of the newborns weighed from 2.5 to 4.5 kg (90.51%). In the family support and social support dimensions, the types of care were more prevalent in the type only by family members and only by other people, at 32.68% and 32.74% respectively; about half of the participants went to work (56.24%); and 98.51% of the participants reported going online.

**Table 1:** Basic information of the participants in this study

Dimension	Variables		Frequency	Component ratio (%)
Demographic characteristics	Residence	Rural	1589	32.84
		Urban	3249	67.16
	Marital status	Unmarried	537	11.10
		Married	4301	88.90
Pre-pregnancy	Pre-pregnancy health status	Good	4468	92.35
		Poor	370	7.65
	Whether trained in pregnancy knowledge	No	2003	41.40
		Yes	2835	58.60
Pregnancy	Whether the pregnancy reaction is intense	No	3186	65.85
		Yes	1652	34.15
	Single or double fetus	Single fetus	4749	98.16
		double fetus	89	1.84
	Sleep	Normal	2856	59.03
		Often insomnia or sleep poorly	892	18.44
		Somnolence	1090	22.53
	Gestational weeks of childbirth	Term birth	4505	93.12
		Preterm birth	310	6.41
		Post-term birth	23	0.47
Postpartum	Depression	No	2610	53.95
		Yes	2228	46.05
	Infant gender	Female	2281	47.15
		Female, female	10	0.20
		Male	2519	52.07
		Male, male	13	0.27
		Male, female	15	0.31
	Infant birth weight	Normal weight	4379	90.51
		Low birth weight	337	6.97
		Overweight	122	2.52
Family support	Type of care	No one care	517	10.69
		Family care	1581	32.68
		Other people care	1584	32.74
		Care by family and others	1156	23.89
Social support	Work	No	2117	43.76
		Yes	2721	56.24
	Internet use	No	72	1.49
		Yes	4766	98.51

### Postpartum depression profiles

In the total sample (n = 4838), 2610 (53.95%) mothers had an EPDS score of < 12 and 2228 (46.05%) had an EPDS score ≥ 12. Therefore, the prevalence of PPD was 46.05%.

Table 2 presents the association of sociodemographic, family support and social support dimensions factors with PPD. Grouped by marital status, the prevalence of PPD was 51.40% in the unmarried group and 45.38% in the married group. The prevalence of PPD was higher in women with excessively low and excessively high family support, women cared by only family members and only others were relatively less affected by PPD. Women with low social support experienced higher levels of PPD and those who had social support experienced less depression, this is related to the shift from family to society of women’s attention.

**Table 2:** Conditions of PPD in different dimensions of sociodemographic–family support–social support (N = 4838)

Variable	Non-postpartum depression	Postpartum depression	*P*
	N (%)	N (%)	
Residence			
Rural	877 (55.19)	712 (44.81)	0.225
Urban	1733 (53.34)	1516 (46.66)	
Marital status			
Unmarried	261 (48.60)	276 (51.40)	*P* < 0.05
Married	2349 (54.62)	1952 (45.38)	
Type of care			
No one care	235 (45.45)	282 (54.55)	*P* < 0.05
Family care	1017 (64.33)	564 (35.67)	
Other people care	844 (53.28)	740 (46.72)	
Care by family and others	514 (44.46)	642 (55.54)	
Work			
No	1090 (51.49)	1027 (48.51)	*P* < 0.05
Yes	1520 (55.86)	1201 (44.14)	
Internet use			
No	22 (30.56)	50 (69.44)	*P* < 0.05
Yes	2588 (54.30)	2178 (45.70)	

Table 3 presents the relationship between factors and PPD throughout pregnancy. The PPD prevalence of the women with good pre-pregnancy health was 44.49% and it was 64.86% of those with poor pre-pregnancy health; the prevalence of PPD was higher in women with pregnancy knowledge training (48.71%), compared with that in those without training. The experience risk of PPD was lower in women with severe pregnancy reactions (43.04%) than in women with normal pregnancy reactions (47.61%); and it was lower in women with normal sleep, relative to those with frequent insomnia or poor sleep (61.32%), and somnolence (51.74%). In infant gender, the prevalence of PPD was particularly high among women with double fetuses that both infant gender were female (80.00%).

**Table 3:** Conditions of PPD throughout the entire pregnancy period (N = 4838)

Variable	Non-postpartum depression	Postpartum depression	*P*
	N (%)	N (%)	
Pre-pregnancy health status			
Good	2480 (55.51)	1988 (44.49)	*P* < 0.05
Poor	130 (35.14)	240 (64.86)	
Whether trained in pregnancy knowledge			
No	1156 (57.71)	847 (42.29)	*P* < 0.05
Yes	1454 (51.29)	1381 (48.71)	
Whether the pregnancy reaction is intense			
No	1669 (52.39)	1517 (47.61)	*P* < 0.05
Yes	941 (56.96)	711 (43.04)	
Single or double fetus			
Single fetus	2561 (53.93)	2188 (46.07)	0.832
Double fetuses	49 (55.06)	40 (44.94)	
Sleep			
Normal	1739 (60.89)	1117 (39.11)	*P* < 0.05
Often insomnia or sleep poorly	345 (38.68)	547 (61.32)	
Somnolence	526 (48.26)	564 (51.74)	
Gestational weeks of childbirth			
Term birth	2435 (54.05)	2070 (45.95)	0.778
Preterm birth	164 (52.90)	146 (47.10)	
Post-term birth	11 (47.83)	12 (52.17)	
Infant gender			
Female	1225 (53.70)	1056 (46.30)	*P* < 0.05
Female, female	2 (20.00)	8 (80.00)	
Male	1362 (54.10)	1157 (45.93)	
Male, male	8 (61.54)	5 (38.46)	
Male, female	13 (86.67)	2 (13.33)	
Infant birth weight			
Normal weight	2364 (53.98)	2015 (46.02)	0.124
Low birth weight	171 (50.74)	166 (49.26)	
Overweight	75 (61.48)	47 (38.52)	

### Binary logistic regression analysis of influencing factors of PPD

**Fig. 1:**
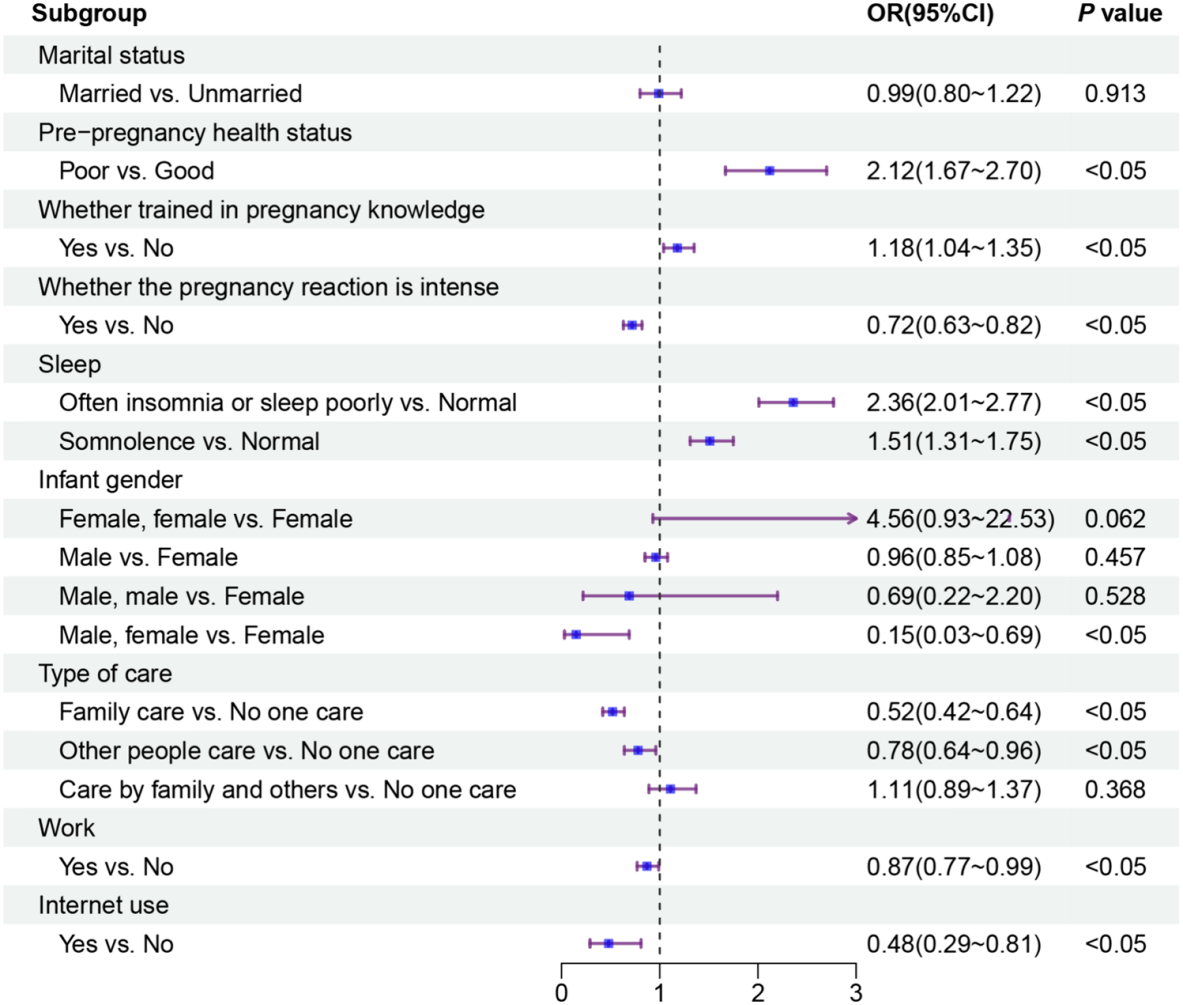
Risk factor analysis for PPD in sociodemography–whole pregnancy–family support–social support dimension

Figure 1 shows the results of binary logistic regression analysis of socio-demographic, whole pregnancy, family support, and social support dimensions with PPD. In the sociodemographic dimension, the marital status of married and unmarried did not show a significant difference. In the pre-pregnancy period, women with poor pre-pregnancy health were 2.12 times more likely to experience PPD than the reference category (good pre-pregnancy health); women who received pregnancy knowledge training (OR = 1.18, 95% CI 1.04–1.35) had a higher experience risk of PPD compared to the reference group. In the pregnancy period, intense pregnancy reactions were a protective factor against PPD for women; having normal sleep was important in protecting women from depression. Compared to the experience risk of PPD in women with normal sleep, those who had often insomnia or sleep poorly were 2.36 times more likely to occur, and those who had somnolence were 1.51 times more likely to occur. In the postpartum period, the variable of infant gender showed a significant difference between only single female fetus and double fetuses with one male and one female infant, double fetuses with one male and one female infant (OR = 0.15, 95% CI 0.03–0.69) was a protective factor against mothers’ PPD compared to single fetus with female infant. In the family support dimension, being cared only by family members (OR = 0.52, 95% CI 0.42–0.64) and being cared only by other people (OR = 0.78, 95% CI 0.64–0.96) were protective factors for PPD compared to being cared by no one, and women cared by no one were more susceptible to the effects of PPD. The variables in the social support dimension were all significantly different, the women who lack social support were more likely to be affected by PPD, and going to work (OR = 0.87, 95% CI 0.77–0.99), going online (OR = 0.48, 95% CI 0.29–0.81) were protective factors for women’s PPD.

## Discussion

We assessed the prevalence of PPD in Yunnan Province, China, and explored the associations between the full-time chain of pre-pregnancy–pregnancy–postpartum, sociodemographic–family support–social support, and PPD. We also expanded the time chain of PPD research, breaking through the causal mechanism of PPD occurrence beyond just physiological or social dimension. We realised the dynamic capture of PPD under the full-time chain, and mapped the vulnerability characteristics atlas of PPD from a multidimensional perspective. The study revealed a high prevalence of 46.1% for PPD in Yunnan Province, which is significantly higher than global (17.22%) and Chinese regional (21.4%) averages [18, 19]. Similar trends were observed when side-by-side comparing with cities such as Shanghai (23.2%) and Guangzhou (27.37%) [20, 21]. Traditional Chinese cultural beliefs regarding unique family dynamics and gender roles may lead to increased family conflicts and closed social networks [22], while China’s rapid economic growth has escalated life stress and elongated work hours [23]. Yunnan Province’s economy is relatively underdeveloped and the scarcity of healthcare resources led to mental health issues being easily neglected. In this study, the average age of childbearing for women was 34 years old. Considering the higher age of childbearing, concerns over medical risks contribute to an increased psychological burden [24]. These factors all contributed to the severe situation of PPD in Yunnan Province. Based on the full-time chain perspective, the PPD prevalence was higher among mothers with poor pre-pregnancy health (64.86%) and sleep problems during pregnancy (often insomnia, sleep poorly: 61.32%; somnolence: 51.74%); and mothers with double fetuses of one male and one female infant had better mental health status after giving birth. In the multidimensional analysis, only family care and only other people care were positive factors for PPD in the family support dimension; going to work or going online had a protective effect on mothers’ health.

Prevention and treatment of PPD should focus on the whole pre-pregnancy–pregnancy–postpartum period and extend the intervention chain.

The PPD experience risk of women with poor pre-pregnancy health was (2.12 times) higher than women with good. This result is consistent with the findings of Michael W. O'Hara et al. [25]. In the pre-pregnancy period, mothers had a previous depression history or possible comorbidities such as hypertension, diabetes, gynecological disorders, with a low physical health level [26, 27]; and in the postpartum period, they suffered from fatigue, pain in wounds, and weakness [28, 29]. The multiple discomforts superimposition makes mothers more prone to postpartum psychiatric problems, such as anxiety, depression, and despondency. Notably, mothers with pregnancy knowledge training were more likely to experience PPD. The possible reason is the mothers with pregnancy knowledge training were more aware of the psychological and physiological changes, that occur during pregnancy and the postpartum period, and they were more likely to think what may cause unnecessary tension, anxiety, and uneasiness [30, 31]. And this increased the PPD experience risk. During the pregnancy period, often insomnia or sleep poorly/somnolence hurt mothers; Some studies have indicated that mothers who sleep 6 h or less were more likely to experience PPD, and sleeping more than 8 h did not significantly decrease the PPD prevalence [32]. The major neurotransmitter systems in the brain involved in regulating sleep have been linked to the development of psychiatric disorders [33]. Therefore, neurotransmitter imbalances can lead to PPD increase. In the postpartum period, twin births of a male and a female infant were a protective factor compared to having only single female infant. In the context of traditional Chinese fertility culture, family members may show some negative reactions to female infants’ birth, that may result in less support for mothers giving birth to a female fetus; whereas a preference for male fetus may be communicated to mothers, and ease their postpartum stress [34]. Additionally, lower marital satisfaction following the birth of female fetus may explain for the increased risk of PPD among the mothers with female fetus. Family members should be well-informed about pregnancy-related matters and offer psychological support to pregnant women. mothers need to maintain a healthy lifestyle and engage in activities that alleviate stress. After childbirth, the focus should shift from solely preventing PPD to prevention and treatment. Nursing interventions are provided to mothers without PPD. Receiving prompt follow-up visits and developing personalized treatment plans is crucial for individuals who have suffered from PPD.

The prevention and treatment of PPD are inseparable from the dual support of family and society, with social support playing an increasingly prominent role.

In terms of family support, only family care and only other care were protective factors of PPD. Family care is the main resource of family support [35]. Family not only provides tangible support such as material and financial support, but also offer mental support from family members, especially husbands, which greatly enhances mothers’ self-esteem and self-confidence, alleviating tension and stress during various pregnancy stages. In addition to family members, medical personnel, friends and colleagues also influence mothers by providing information support, emotional accompaniment and value recognition [36, 37]. In terms of social support, going to work or going online can reduce the risk of PPD, it not only increases mothers’ self-efficacy, but also provides the understanding and appreciation they need as they transition to motherhood [38]. The social climate in social networks reflects a stigma associated with mental health problems, which acts as a barrier to seeking professional help for mothers. However, positive social behaviours can enhance cognitive abilities of mental health problems, overcome perceptual barriers and help-seeking intentions, and reduce the stigma of mental illness [39]. Family support for mothers should encompass emotional, informational, material, and interactive aspects, focusing on recognizing emotional shifts, providing comfort, sharing childcare knowledge, and ensuring effective communication. Communities ought to deliver holistic primary health care, including early detection, education, and postpartum support. The government should consider establishing childcare allowances and creating job opportunities to facilitate mothers’ societal reintegration post-birth.

## Data Availability

The data that support the findings of this study are available on request from the corresponding author.
